# 

**Published:** 1875-02-01

**Authors:** 


*ORIGINAL COMMUNICATIONS:*

Balanitis. By F. K. Bailey, M.D. ........ 57
Hernia of the Whole Bowel. By H. H. Briggs, M.D. ........ 62

*CLINICAL REPORTS:*

Pericarditis, with Broncho-Pneumonia. By N. S. Davis, M.D. ........ 63

*TRANSLATIONS:*

Progress of Medical Science in Germany. By E. J. Doering, M.D. ........ 67

*EDITORIAL DEPARTMENT:*

Idiotic and Feeble-minded Children ........ 70
Health of Public Reformatories ........ 71

*SOCIETY REPORTS:*

Transactions of the Chicago Society of Physicians and Surgeons. Regular Meeting, Jan. 11, 1875 ........ 71
Will County Medical Society ........ 73

*GLEANINGS FROM OUR EXCHANGES:*

On the Hand-feeding of Infants. By Eustace Smith, M.D. ........ 74
The Physiology of Versification—Harmonies of Organic and Animal Life. By Oliver Wendell Holmes, M.D. ........ 79
A Case of Supposed Hydrophobia ........ 82
Paralysis of the Accommodation of the Eye, following an Attack of Diphtheria. By P. A. Callan, M.D. ........ 85

